# Women’s Empowerment and Livestock Vaccination: Evidence from Peste des Petits Ruminants Vaccination Interventions in Northern Ghana

**DOI:** 10.3390/ani12060717

**Published:** 2022-03-12

**Authors:** Immaculate Omondi, Alessandra Galiè, Nils Teufel, Agnes Loriba, Eunice Kariuki, Isabelle Baltenweck

**Affiliations:** 1 Policy, Institutions and Livelihood, International Livestock Research Institute, Nairobi 30709-00100, Kenya; a.galie@cgiar.org (A.G.); n.teufel@cgiar.org (N.T.); e.kariuki@cgiar.org (E.K.); i.baltenweck@cgiar.org (I.B.); 2 CARE International Ghana, Accra CT 2487, Ghana; agnes.loriba@care.org

**Keywords:** women’s empowerment, WELI, PPR, vaccination, Ghana, knowledge

## Abstract

**Simple Summary:**

Small ruminants (goat and sheep) are key livestock species in supporting women’s empowerment (WE) in low- and middle-income countries. Animal vaccines are essential for livestock productivity, hence an important means to support WE. WE is, in turn, important for animal vaccine adoption. Little is known, however, of how WE is associated with animal vaccination for women-controlled livestock assets (e.g., goats and sheep). Our analysis explores the link between domains of WE and knowledge of, access to, and use of peste des petits ruminants (PPR) vaccines. Such knowledge can help inform the design of livestock vaccine systems that are better able to reach women and support their empowerment. Using a partial least squares structural equilibrium model (PLS-SEM), we analyzed data collected using the Women’s Empowerment in Livestock Index (WELI) tool from goat keepers in Northern Ghana, which included a module on the PPR vaccine. We found a strong direct positive association between women and men’s knowledge about animal health and PPR vaccination and a strong indirect positive association between access to PPR vaccines and empowerment. Moreover, women and men goat keepers differed in the dimensions of empowerment that the PPR vaccine facets were strongly associated with—asset ownership and input into decisions concerning livestock was significant for women but not for men. Consequently, policy and actions towards enhancing women’s asset ownership, input into decisions about livestock production, knowledge of animal health and vaccines, and access to vaccines are important in designing effective and equitable livestock vaccine systems.

**Abstract:**

Healthy livestock provide meaningful opportunities to enhance women’s empowerment (WE) in low- and middle-income countries. Animal vaccines are important to keep livestock healthy and productive. However, gender-based restrictions limit women’s access to animal health services, thereby affecting the potential of livestock to enhance their empowerment. While growing empirical evidence reveals that women-controlled livestock (e.g., small ruminants) have important implications for WE and support better household nutrition outcomes, little empirical evidence exists from rigorous analyses of the relationship between WE and animal vaccines for women-controlled livestock species. Our analysis explores the relationship between WE and involvement with PPR vaccination in Ghana. Data collected using the Women’s Empowerment in Livestock Index (WELI) tool from 465 women and 92 men farmers (who keep goats) from northern Ghana, and analyzed using PLS-SEM, revealed a significant direct positive association between knowledge about animal health and PPR vaccines and a significant indirect positive association between access to PPR vaccines and empowerment. The empowerment of women goat farmers, as revealed by our model’s results for the relationship between empowerment and vaccine facets, was significantly represented by asset ownership and input into decisions concerning livestock. These study results reveal important considerations in designing effective and equitable livestock vaccine systems.

## 1. Introduction

Livestock, as a store of wealth as well as a source of income and nutritious food, can provide meaningful opportunities to support livelihoods in low- and middle-income countries, particularly for rural women [1]. Women-controlled livestock, in particular (often small species, such as small ruminants and poultry), have been shown to support the livelihoods of women, their empowerment and their households’ nutritional outcomes [2,3]. Consequently, targeting women through interventions that support livestock management can generate greater empowerment and reduce inequality gaps [4].

When engaging with the concept of empowerment, we adopted a definition of empowerment as a “multi-dimensional social process that helps people gain control over their own lives” [5]. It is the process of enhancing the capacity of individuals or groups to make choices and to transform those choices into desired actions and outcomes. A number of studies have shown that sustainable development is impossible without WE and gender equality [5]. The pathways toward women’s empowerment (WE) include, for example, reducing female–male differences in resource access, increasing women’s participation in labor markets, leveraging female–male differences to increase women’s relative decision-making authority related to agricultural management and production, and increasing productivity [6]. Knowledge about, access to, and use of animal vaccines is one of the elements of agricultural management for production and increasing productivity.

**Figure 1:**
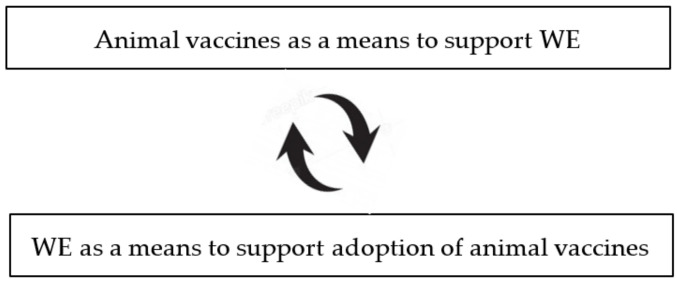
Conceptual association between WE and animal vaccines.

Veterinary care and services are essential to protect animal health and enable a more efficient production of animal-source foods, and are, therefore, an important means to support WE through livestock [7]. However, gender-based restrictions affect women’s access to animal vaccines and thus the potential of livestock to support their empowerment and livelihoods. Restrictions on women’s mobility, for instance, make it difficult for women livestock keepers to access vaccines, extension services, information and markets, and, consequently, to raise healthy and productive livestock for sustainable livelihoods and empowerment [8]. If women were more empowered, it could be expected that households may have a higher propensity towards adopting new agricultural technologies and approaches [9]. Therefore, while access to animal vaccines is an important means to support WE, WE is, in turn, important for animal vaccine adoption because empowered livestock keepers are more likely to adopt vaccines. Figure 1 is a simplification of the relationship between WE and animal vaccines using one of the pathways, efficient production of animal-source foods.

In two study sites in Kenya’s Uasin Gishu County (mixed crop–livestock production system) and Kajiado County (transhumant pastoral system), cattle vaccinations led to reduced livestock deaths and increased herd sizes [8]. However, because women performed most of the cattle rearing activities while men controlled cattle sales, women’s workload increased while men benefitted from controlling the increased animal sales income [8]. Such dynamics have been seen to negatively affect the willingness of women livestock keepers to adopt new animal vaccines [8]. Therefore, it is important to appreciate the links between animal vaccines and WE for more effective and equitable vaccine systems and more sustainable and equitable livestock development.

In the literature, we find studies that have explored vaccinations in different ways, for instance, gendered barriers to livestock vaccine uptake [10], how gender and other social factors affect the provision and utilization of vaccines [11], the effects of livestock vaccination on achieving the Sustainable Development Goals (SDGs) [12], perceptions and the impact of infection and treatment method (ITM) vaccinations on the livelihoods of men and women [8], and the relationships between livestock vaccinations and livestock disease-related outcomes among others [13]. We find no study that has explored the nexus between WE and livestock vaccination. Consequently, there is limited empirical evidence from rigorous analyses to examine how WE is influenced by or influences animal vaccination, particularly for women-controlled livestock assets, as conceptualized in Figure 1. This study explored the links between WE and PPR vaccination in northern Ghana, with the goal of informing the design of livestock vaccine systems that are more effective and equitable.

In many parts of the developing world, sub-Saharan Africa in particular, women commonly own and manage livestock. They play key roles in livestock value chains by providing much of the required labor. Often, they have more authority over small stock, especially goats and chickens, compared to large livestock, e.g., cattle [14,15,16,17,18]. In Ghana, poor households, particularly the women within them, often depend on chickens and small ruminants (especially goats) for income, especially in times of risk [19] (related to yield-reducing factors on alternative livelihood options, such as weather and climate shocks, pests and diseases, price shocks, and human health issues or personal relationships that affect the farm or farm household [20]). Yet small ruminant production, especially under traditional systems, is limited by high mortality and morbidity rates [21].

Peste des petits ruminants (PPR), otherwise known as “goat plague”, is an acute, highly contagious and transboundary viral disease of sheep and goats that causes high morbidity and mortality, with major constraints in the productivity of small ruminants in certain parts of the world [22]. PPR, one of the priority animal diseases whose control is only possible through vaccination, is considered important for poverty alleviation in Africa and Southern Asia [22]. It is a devastating livestock disease owing to its high mortality. For instance, during the 2006–2008 PPR outbreak in northern Kenya (Turkana District), over a million animals perished, resulting in production losses estimated at USD 2.4 million [23]. In Ghana, it is one of the most devastating livestock diseases that limits the small ruminant industry and causes substantial economic losses to small ruminant farmers, particularly women, who are the majority of goat keepers [24,25].

Although an effective vaccine against PPR is available in Ghana, the vaccine delivery system tends to reach men farmers rather than women. For instance, Enahoro et al. (2021) [26] found that women experienced limited access to veterinary drugs and vaccines (and other inputs) because of their limited mobility, and that more women farmers, compared to men, had limited formal education and hence they suffered more constraints attributed to poor access to veterinary extension. This observation is corroborated by empirical evidence of women farmers’ significantly lower access to technology and information outreach compared to men supplied by Meinzen-Dick et al. (2010) [27] and the World Bank and IFPRI (2010) [28]. Moreover, in central Ghana, access to resources, such as land, agricultural extension services, and information, are gendered and intersect with other factors, such as education and sociocultural norms, that shape access to and control over resources [29]. Some sociocultural factors prevent women from directly having access to extension services (e.g., extension officers being mostly men, women are not expected to spend time getting advisory service from men) [29]. The limitations in access to technology, information outreach, and sociocultural norms make it difficult for women to access the PPR vaccine and related information, arguably reducing the potential of WE through small ruminants. The gender gap in education in Ghana is also still pronounced, with women having comparatively lower education levels than men [29,30].

Current PPR eradication efforts, particularly strategies for strengthening veterinary services [31], provide a distinct opportunity to serve as a platform for the application of lessons learned from past vaccination initiatives and for investigating additional questions that will improve future vaccination strategies [32]. Gendered factors play an important role in determining individuals’ abilities to operate in the vaccine distribution system and/or access livestock vaccines. On the other hand, an efficient and equitable livestock vaccine distribution system that focuses on diseases affecting women-controlled livestock (such as poultry or small ruminants) may benefit smallholder farmers, women especially, by supporting their livelihoods given their greater economic dependence on these livestock species [11]. Advancing these two pathways requires targeting the priorities of men and women and local gender norms, as well as the possible impacts of interventions on men, women, and gender dynamics as a step towards designing gender-responsive animal health delivery systems [33]. Consequently, understanding the association between WE and PPR vaccination facets helps in advancing the understanding of possible impacts of interventions on men and women. A PPR eradication strategy that responds to gender-based constraints and opportunities and intentionally supports WE is critical to finding the most effective approach for the global eradication of PPR [32]. The joint Food and Agriculture Organization of the United Nations/World Organisation for Animal Health (FAO/OIE) global PPR eradication strategy recognises that while eradication of PPR is the ultimate goal, to be attained by the year 2030, the strategy cannot be a “stand-alone” activity, hence its additional focus on strengthening veterinary services and improving the prevention and control of other major diseases of small ruminants [31]. Since 2015, the global eradication efforts (disease-control strategies) have seen a decline in the number of global PPR outbreaks in 2019 (which fell by 67% from 3688 in 2015) [34]. These PPR control strategies, particularly vaccination campaigns and vaccine distribution strategies, still require scientific evaluation [34]; consequently, a rigorous analysis that informs the design of a gender-responsive animal health delivery system would contribute towards the strengthening of veterinary services.

This paper provides a better understanding of the relationship between the empowerment of women farmers and their engagement with PPR vaccination. When focusing on vaccination, factors such as access to vaccines (encompassing “physical availability of a vaccine in a given location”; “physical ability of an individual to get a vaccine”, and “ability of an individual to purchase it”), and vaccine demand and use (implying “being aware of the existence of a vaccine” and “wanting to use the vaccine”) are important in assessing vaccine supply chain links [35]. Similar factors, i.e., knowledge of animal health and the availability, affordability, and quality of animal health inputs and services, have also been used to assess the delivery of animal health services (including vaccinations) [36]. Based on this evidence, this study covered three distinct facets of PPR vaccination: “knowledge about animal health and vaccines”, “access to PPR vaccines” (physical access and affordability), and “participation in vaccination” (involvement in getting or purchasing vaccination services, paying for vaccinations, and having livestock vaccinated).

In this study, we operationalized WE as an intrinsic, instrumental, and collective agency by adopting the indicators included in the Women’s Empowerment in Livestock Index (WELI) tool. Based on a cross-sectional data set, we analyzed the association between the three selected facets of PPR vaccination and the empowerment domains included in the WELI. The analysis does not allow the determination of causal links, including their direction, which would indicate whether WE has an impact or is a determining factor in increased vaccine adoption. Nevertheless, evidence of the relative importance and interdependence of the vaccination facets vis-à-vis WE will inform strategies for further research and some initial recommendations for a more gender-responsive animal vaccination system.

This paper is structured as follows: Section 2 details the study methodology and includes a review of the empirical method applied and details on data sources employed in the study. Section 3 interprets the main results of the model. Section 4 presents the main conclusions and implications for policy and strategic action based on empirical results.

## 2. Materials and Methods

The study used the WELI tool [37] to measure the empowerment of women in 465 rural goat-keeping households and 92 men from a sample of the same households in two districts in northern Ghana, Bawku West District and Pusiga District in the Upper East Region. The WELI tool is designed to measure livestock interventions’ impact on WE. It focuses specifically on key decisions in livestock production, such as animal health, breeding, and feeding, as well as the use of livestock products, such as animal-sourced food processing and marketing. It also includes questions on crops. The tool is aligned to the project-level Women’s Empowerment in Agriculture Index (pro-WEAI) [38], a tool that captures aspects of WE relevant to the outcomes of crop agricultural development, in particular. WELI uses data from both male and female respondents to make direct comparisons between men and women in the same household and separately diagnose the aggregate sources of disempowerment for men and women [39]. The WELI version utilized in this study included a module that focused on PPR vaccination (details below). The study applied partial least squares structural equation modelling (PLS-SEM) to analyze the relationship between vaccine facets and empowerment.

The study used data from the “Transforming the vaccine delivery system in Ghana: identifying approaches that benefit women” (“WomenRear”) project’s 2021 face-to-face survey of livestock-keeping households, particularly goats and poultry farmers from two districts, Bawku West and Pusiga, in the Upper East region of Ghana. This four-year research for development project, implemented from 2019, is led by Care International [40] in collaboration with the International Livestock Research Institute (ILRI) and CowTribe [41]. The current vaccine delivery system in Ghana is led and implemented by the government, i.e., the Veterinary Department of the Ministry of Food and Agriculture. The department stocks PPR vaccines at district level for use during vaccination campaigns whenever there is an outbreak or whenever individual farmers demand it through government animal health workers. Mass or targeted PPR vaccination campaigns are usually implemented by animal health workers in the form of door-to-door delivery or at specific small ruminant vaccination locations. When the campaigns are organized by the government, vaccinations are free of charge to the farmers. However, these free vaccination campaigns have been rare in the study area in recent years, prompting farmers to individually search and pay for the vaccinations offered at a fee by the government and private animal health workers. Most, if not all, animal health workers are men. The vaccine delivery system results in vaccines being more accessible to male farmers than to female farmers, largely due to the current gender norms and inequalities (e.g., women’s mobility constraints, financial constraints, and social norms that govern male–female interactions). Consequently, the project’s aim is to develop evidence and recommendations for a gender-responsive animal vaccination system in Ghana with a focus on PPR and Newcastle disease.

The project is designed to compare gender accommodative approaches (GAA) and gender transformative approaches (GTA) by adapting CARE International’s Gender Transformative Farmer Field and Business School approach to facilitate the sustained involvement of women in livestock vaccination. Gender-transformative interventions aim to transform and change existing gender norms and barriers while gender-accommodative interventions specifically target women but do not engage with the system as a whole [42]. Both approaches address the immediate practical barriers to accessing vaccines as well as gender-based barriers, such as gender norms’ effects on decision-making and the mobility of women.

### 2.1. Study Area

The Upper East Region is one of the five northern administrative regions of Ghana. Much of northern Ghana falls within the savannah vegetation belt. Rainfall is modest in many parts of the area but allows for the cultivation of cereal crops and legumes. Agriculture and agro-based industries are the mainstay of the people of this zone, with animal husbandry traditionally being an integral feature of agricultural production systems [43]. Cattle, sheep, goats, and local poultry (chickens, ducks, guinea fowl, pigeons, turkeys, and doves) are the major livestock species raised in the area [44]. Men predominantly keep cattle while women mainly keep poultry, sheep, and goats, except in some communities where women do not keep any livestock at all [44]. In northern Ghana, livestock, especially small ruminants (i.e., sheep and goats) are kept by over 80% of smallholder farmers [45] who are considered to be the largest and most vulnerable (to risks associated with crop and/or market failure) component of the rural sector [46]. Fewer households keep sheep compared to goats (only 3 out of 10 households keep sheep) [45]. In the study districts, goat and pig production are second in economic importance after poultry, particularly among women and women-headed households [44]. The major challenges to livestock health in the region include Newcastle disease (NCD) in poultry and pestes des petits ruminants (PPR) in small ruminants [44].

### 2.2. Sampling

The study respondents were randomly selected using a multi-stage sampling process. First, all villages/communities in the two study districts with active village savings and loans associations (VSLAs) were listed. Based on discussions with the local partners and stakeholders, women with access to VSLAs were assumed to be more likely to be able to afford vaccines. Since the WomenRear project timeline did not allow for starting new VSLAs, the project focused its activities on villages/communities with active VSLAs and did not establish new VSLAs in villages/communities where none existed. In each of the two districts, five communities were randomly selected from this list for a GAA intervention only and five communities for a GTA intervention in combination with the GAA intervention, resulting in a total of 20 selected communities. However, in Pusiga District, two pairs of communities were reassigned between groups to reduce the risk of spillovers between the treatment groups due to their proximity to communities in the other group.

All households engaged with VSLAs in the selected communities were listed to generate a household sampling frame for the survey. Collected variables, for generating the sampling frame, included those for identification (name of household head, etc.) as well as variables for inclusion (VSLA membership, livestock ownership). The listing yielded 507 households from Bawku West and 639 from Pusiga. Most of the households listed in the sampling frame were male-headed with only one wife; only 7% and 3% of households in Bawku West and Pusiga, respectively, included more than one wife. For the survey, only first wives were targeted as respondents in polygamous households to reduce potential additional sources of variation in empowerment. Subsequently, 8.3% and 2.2% of first wives in Bawku West and Pusiga, respectively, were excluded from the sampling frame because they were keeping neither chicken nor goats (the target species). Only one household listed in the sampling frame was woman-headed. Since gender roles and empowerment characteristics in such households are different from coupled households, this household was also excluded. As a result, 91.7% of households in the sampling frame from Bawku West and 97.8% from Pusiga were eligible for selection.

Since the empowerment index was the main evaluation criterion of the project, the estimation of the required sample size for the quantitative household survey was based on power calculations using data from a previous WELI survey conducted in Tanzania [47]. The results of the power calculations showed that an unadjusted sample size of 114 households allowed a minimum detectable difference (mdd) of 20% of the overall WELI index mean, while for the indicator on production decisions 120 households would allow for the detection of a difference of 35% of the mean. These detectable differences were acceptable for the envisioned analysis. Therefore, we arrived at a sample size of 125 households per group (two GTA/GAA intervention groups per district) for the household survey, resulting in a total sample of 500 households for the two treatment groups in the two project districts.

WELI can capture the relational nature of empowerment by interviewing both a woman and a man within a household [47]. Consequently, in addition to interviewing one woman from each of the sampled households, the male household heads of a subset of the sampled households were also interviewed using the same WELI tool that was used to gather quantitative data from the sampled women. The purpose of including men was to estimate the gender parity index (the difference between women and men empowerment indices) in a bid to explore patterns in gender relations within the surveyed households. Since the aim was not to test whether the project interventions will affect gender parity, a 40% mdd relative to the mean, assuming a coefficient of variation of 1, was deemed acceptable. This would require a group size of 24 households. Therefore, 25 households were selected for this sub-sample per district and treatment, resulting in a total of 100 households in which men household heads were also interviewed. The data from the sampled women and men respondents was deemed to be representative of the project districts due to the weighted random selection of communities and the simple random selection within the selected communities. The final data set generated by the household survey consisted of interview records from 493 women and 97 men.

### 2.3. Data and Variable Description

The WELI-related variables in the collected data are aggregated to 13 indicators (see Table A1 in Appendix A), with which empowerment index values are determined. The vaccine module added to the WELI tool aimed at understanding the context and dynamics of various vaccine dimensions in the study area. The variables from the vaccine module used in this study are described in Table 1. In this study, we only analyzed data from goat-keeping households, i.e., 465 women respondents and 92 men respondents.

This study focused on the relationship between vaccination facets (the exogenous latent variables) and concepts involving resources, agency, and achievements in the livestock sector (the endogenous latent variables). Primary endogenous latent variables in PLS-SEM act as dependent variables (i.e., variables that are explained by the relationships contained in the model). The primary endogenous latent variables in our PLS-SEM analysis were the WELI indicators. WELI encompasses three dimensions: resources (entitlements and access to physical, human, and social capital that boost the capacity to exercise choices), agency (progressions in abilities to make decisions) and achievement (welfare outcome) [47,48]. The three dimensions measure WE across three domains: intrinsic agency (power within), instrumental agency (power to), and collective agency (power with), hence abbreviated as 3DE (three domains of empowerment) [39]. The variables from the vaccine module (Table 1) entered the PLS-SEM analysis as independent variables.

### 2.4. Analytical Method

#### 2.4.1. WELI Construction

First piloted in 2015 [47], WELI has been increasingly aligned to pro-WEAI [38], hence its construction follows the procedure outlined by Malapit et al. (2019) [39]. WELI’s 3DE index values are determined by aggregating 13 indicators. A detailed description of the indicators (namely, autonomy in income, self-efficacy, attitudes about intimate partner violence against women, respect among household members, input in productive decisions—general, input in productive decisions—livestock, ownership of land and other assets, access to and decisions regarding financial services, control over use of income, work balance, ability to visit important locations, group membership, membership in influential groups) can be found in Table A1 in Appendix A. These indicators differ from pro-WEAI only in that the questions that contribute to most of them are focused on livestock and that there is one additional indicator (i.e., input in livestock production decisions). The method of scoring used is analogous to standard methods for computing the pro-WEAI and other variants of the index. It is based on data obtained from individual respondents from households that derive part of their livelihood from livestock production. To determine whether an individual has achieved a minimum level of empowerment for a particular indicator, questions contributing to an indicator are considered together, sometimes in several steps. Each respondent is classified as either adequate (=1) or inadequate (=0) with respect to a given indicator by comparing their responses to the survey questions with a given threshold (Table 2). A respondent’s empowerment score is simply the weighted average of her/his adequacy scores in the 13 indicators (all weighted 1/13). If her/his score is 75% or higher, s/he is classified as empowered.

#### 2.4.2. Conceptual and Empirical Model

**Figure 2:**
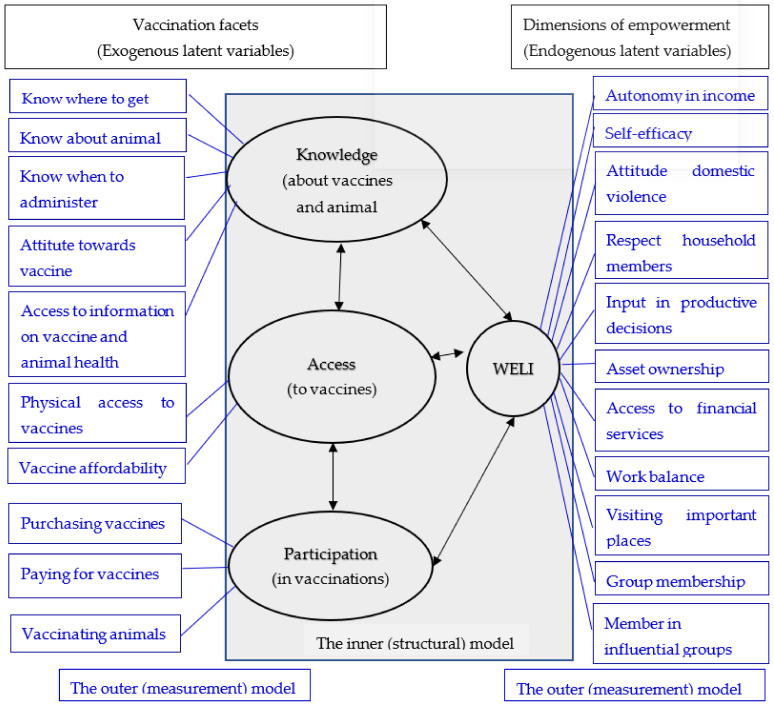
Conceptual model for evaluating the relationship between empowerment dimensions and vaccination facets.

Three facets of vaccine interventions (knowledge and attitude about vaccines and animal health, access to vaccine and vaccine information, and participation in vaccinations) were used as the exogenous latent variables and WELI (defined by its 13 indicators) was the endogenous latent variable in the estimation model, as depicted in Figure 2. We hypothesize that the three vaccination facets (knowledge, access, and participation) are associated with the empowerment of farmers and that the vaccination dimensions are interrelated.

Since the 13 indicators that contribute to WELI index values are correlated, they are challenging to analyse. However, aggregating these indicators into higher order constructs (HOC) in structural equation models (SEM) makes the phenomena being observed easier to understand [49]. SEMs enable researchers to simultaneously model and estimate complex relationships among multiple dependent and independent variables; the concepts under consideration are typically unobservable and measured indirectly by multiple indicators [50]. In other words, this second-generation multivariate data analysis technique, SEM, enables researchers to answer a set of interrelated research questions in a single, systematic, and comprehensive analysis by modeling the relationships among multiple independent and dependent constructs simultaneously [51], unlike first-generation regression models, such as linear regression, LOGIT, ANOVA, and MANOVA, which can only analyze one layer of linkages between independent and dependent variables at a time [52]. While SEM is similar to multiple regression in the sense that both techniques test relationships between variables, SEM is able to simultaneously examine multi-level dependence relationships [53] where a dependent variable becomes an independent variable in subsequent relationships within the same analysis [54] as well as relationships between multiple dependent variables [50]. Therefore, one no longer differentiates between dependent and independent variables [55] but distinguishes between the exogenous and endogenous latent variables, the former being variables which are not explained by the postulated model (i.e., act always as independent variables) and the latter being variables that are explained by the relationships contained in the model [56]. SEM permits the simultaneous analysis of multiple linear regressions between the independent variables, a multiple path analysis, the assessment of the direct and indirect effects, and estimates the fitness of the overall model, which is not feasible in a traditional regression analysis method [57]. SEM can also provide measures of fit to assess the entire model [58].

There are two approaches to estimating the parameters of SEM, namely, the covariance-based approach and the variance-based (or components-based) approach [55]. Variance-based SEM (VB-SEM) determines construct scores as linear combinations of observed variables in a way that maximizes certain criteria of interrelatedness [59]. Covariance-based SEM (CB-SEM) attempts to minimize the difference between the sample covariances and those predicted by the theoretical model [55]. All variance-based SEM techniques (for instance, regression based on sum scores or principal components, partial least squares path modeling (PLS), and generalized structured component analysis) approximate latent variables using linear composites of observed variables [60]. CB-SEM and partial least squares SEM (PLS-SEM, also called PLS path modeling) are the two popular methods that dominate SEM in practice [50]. CB-SEM is primarily used to test theories and their underlying hypotheses by determining how closely a proposed theoretical model can reproduce the covariance matrix for an observed sample dataset [50]. PLS-SEM is an alternative to CB-SEM, which provides researchers with more flexibility in terms of data requirements, model complexity, and relationship specification [61].

PLS-SEM is useful because the non-parametric approach places fewer limitations on sample size and data distribution, compared to CB-SEM. It is appropriate to build theories and explore models with various cause–effect relationships [62,63,64]. PLS-SEM enables researchers to analyse both the measurement and structural models, while allowing the incorporation of both unobserved (construct/latent factors) and observed variables in the same model [65,66]. This analytical method also handles errors of measurement within exogenous variables having multiple indicators by the usage of confirmatory factor analysis (CFA) [67]. PLS-SEM requires the computation of construct scores for each latent variable in the path model [68]. The three approaches to model hierarchical latent variables in PLS-SEM that have been proposed in the literature are the repeated indicator approach [69,70], the sequential latent variable score method or two-stage approach [71,72], and the hybrid approach [73]. The advantages and disadvantages of each of the three approaches are discussed in Becker et al. (2012) [68].

Since PLS-SEM is the preferred method when the study object does not have a well-developed theoretical base, particularly when there is little prior knowledge of causal relationships (i.e., the emphasis here is on the explorations rather than confirmations [74]), we use PLS-SEM to examine the relationship between WE and farmer-level vaccination facets (i.e., farmer’s access to vaccines, farmers’ knowledge about animal health and vaccines, and farmers’ participation in vaccinating their animals). We test the hypotheses on the relationship between the vaccination facets (variables presented in Table 1) and all the 13 empowerment indicators from WELI (see Table A1 in Appendix A, as conceptualized in Figure 2. PLS-SEM relies on two different conceptual models: (i) a measurement model which assesses validity and reliability between the observed variables and the latent causal constructs; and (ii) a structural model which tests the significance of the relationship among the latent constructs, the predictive power of different variables, and the variance of the endogenous variables [58] (illustrated in Figure 2). To achieve the study objectives, SmartPLS 3 (SmartPLS GmbH, Bönningstedt, Schleswig-Holstein, Germany) [75], one of the prominent software applications for PLS-SEM [76], was used to estimate the PLS-SEM model. This study followed a two-stage path aligned with previous studies [48,77,78]. Since PLS-SEM modeling does not provide any global goodness-of-fit criterion, the two-step process encompasses a criterion to assess partial model structures which involves (i) the assessment of the outer model and (ii) the assessment of the inner model [62].

## 3. Results

### 3.1. Descriptive Analysis—Sociodemographic Characteristics of the Study Participants

Data collected using the WELI tool from sampled respondents (465 women and 92 men goat keepers) were analyzed (frequencies and means) to provide an overview of household characteristics (Table 2). The average age of women farmers was 44 years, while men were approximately 46 years old. The women had significantly smaller household sizes (number of people living in their households) compared to their male counterparts. Asked to indicate the livestock species that they felt to be important for their households and for their own livelihood, respondents indicated small ruminants as the most important. Comparatively more women than men regarded small ruminants as important for their household’s livelihood. In terms of their own livelihood, both men and women regarded small ruminants and chicken as the two most important livestock species. The results (Table 2) concur with the literature presented in Section 2.1 in terms of the species kept.

### 3.2. Analytical Model Reliability and Validity

In surveys where data is collected through self-reported questionnaires, common method bias (a phenomenon that may arise from the possibility that instructions provided by a researcher administering a questionnaire influence the answers provided by different respondents in the same general direction, hence causing the indicators to share a certain amount of common variation [77]) may arise. Common method variance (CMV) needs to be examined, particularly when both the predictor and criterion variables are obtained from the same individual [78]. Harman’s single factor test is one of the widely used tests of CMV, which entails creating a model with one single latent variable and conducting a composite-based or a factor-based analysis [77]. Partial correlation procedure and correlation matrix procedure have also been widely used by the researchers to address the issue of common method bias [79]. Although extensively used, these tests are not sufficient to control common method bias because they can only test the presence or absence of CMV [79]. Using the correlation matrix procedure, we found no existing CMV in our analyses, hence no need to apply further remedies for common method bias.

Further tests were conducted to assess the model constructs’ accuracy, consistency, and reproducibility. Convergent validity, the extent to which the construct converges in order to explain the variance of its indicators, is evaluated using the average variance extracted (AVE) for all indicators of each construct [80]. Convergent validity is achieved when a set of indicators of a construct converge or represent a single underlying construct [58]. The minimum acceptable AVE (defined as the grand mean value of the squared loadings of the indicators associated with the construct) is 0.50—an AVE of 0.50 or higher indicates that the construct explains 50% or more of the indicators’ variance that make up the construct [80]. The reliability of the variables was tested using Cronbach’s Alpha (CA) and Composite Reliability (CR). CA, expressed as a number between 0 and 1, provides a measure of the internal consistency of a test or scale. Internal consistency describes the extent to which all the items in a test measure the same concept or construct [81]. CR is also an internal consistency measure that tests the overall reliability of a collection of heterogeneous but similar items [82].

Initially, the overall sample was assessed and items having factor loadings that were smaller than 0.500 were discarded. The results for reliability and validity along with the factor loadings for the remaining items are presented in Table 3 for both women and men respondents. Most of the CA values and CRs were higher than or close to the recommended value of 0.700 (the raw mean inter-item correlation, also used as a statistical marker of internal consistency, fell in the acceptable range of 0.15–0.50), indicating consistency in internal reliability. Convergent validity was also assessed by assessing average variance extracted (AVE). All the values exceeded the threshold of 0.5 [58]. Discriminant validity, a metric that measures the extent to which a construct is empirically distinct from other constructs in the structural model [80], was assessed through cross-loadings. Multicollinearity was also assessed, with the value of each indicator’s variance inflation factor (VIF) less than 5. It was observed that all the factor loadings are greater than their cross-loadings, which is a sign of discriminant validity. Discriminant validity was also tested using the criterion suggested by the Fornell–Larcker criterion and the heterotrait–monotrait method (HTMT).

### 3.3. Model Results: The Association between PPR Vaccine Facets and Empowerment

**Figure 3:**
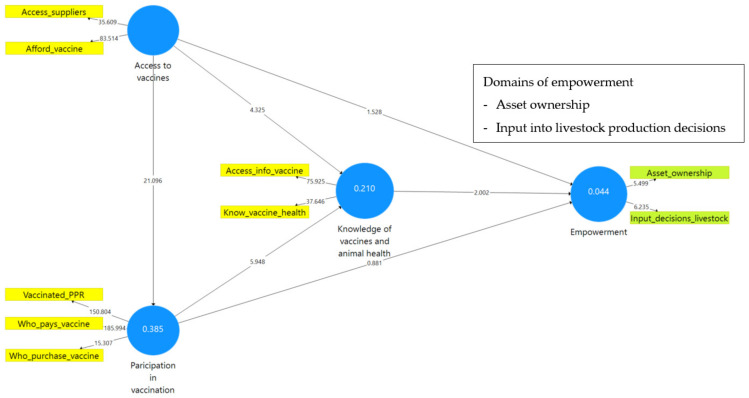
PLS-SEM model results from women respondents.

**Figure 4:**
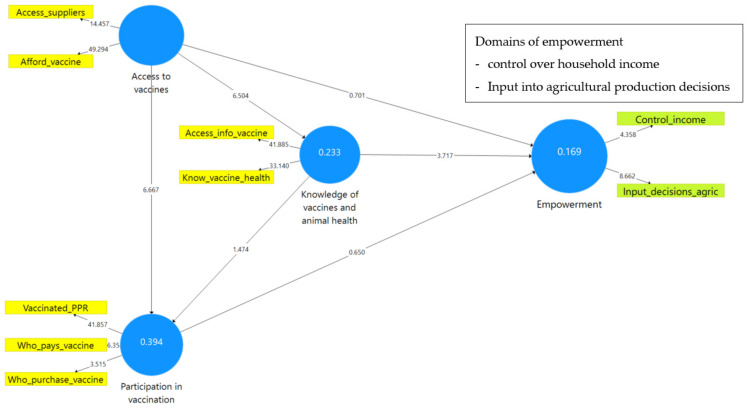
PLS-SEM model results from men respondents.

Figure 3 shows the results of the PLS-SEM model for women respondents while Figure 4 shows the results of the same model from men respondents. In PLS-SEM, the path coefficients and the values of the R-square (R^2^) scores for the endogenous variables are used to explain the strength of the structural model; the coefficients are used to evaluate the implication and significance of the relationships [48]. R^2^ represents the combined effects and variance of all exogenous constructs linked to the endogenous construct; the effect size, f^2^ (F-squared), evaluates an explanatory variable’s considerable impact on an endogenous variable [58]. F-squared (f^2^) for each path model can be determined by calculating Cohen’s f^2^ (computed by noting the change in R^2^ when a specific construct is eliminated from the model) [83]. The endogenous variable, the empowerment index in this study, has an R^2^ value of 0.044 for women respondents (Figure 3) and 0.169 for men respondents (Figure 4). This implies that about 4.5% and 17% of women’s and men’s empowerment, respectively, was accounted for by knowledge of vaccines, access to vaccines, and participation in vaccinations.

To evaluate the significance and relevance of the model, the exogenous and endogenous variables’ path coefficient outcomes were assessed using bootstrapping. Bootstrapping examines the coefficient’s statistical significance by computing the coefficient estimates [64]. The explicit latent variable impact on the endogenous variable is shown Table 4 (for women) and Table 5 (for men). For the exogenous variables in this study, the effect size (estimated but not shown in the table) varies from small to large. Table 4 and Table 5 show the direct relationship between empowerment and vaccination constructs with their respective path coefficients for women and men respondents, respectively. The *p*-values in the table are interpreted in the same way as the *p*-values from regression models, for instance, *p* < 0.05 indicates a significant correlation between two variables at 5% significance level.

The model results (Table 4 and Table 5) reveal the variables in each of the vaccine facets that were significant. The “participation in vaccination” variable included whether or not a respondent’s animals were vaccinated, who goes to fetch or receives the vaccinators, and from whose pocket or budget in the household the money used to pay for the costs incurred in the vaccination process comes from. “Access to vaccine” focuses more on the limitations in acquiring the vaccines, such as accessibility (e.g., distance to the supplier or frequency of availability in the suppliers’ location of operation) and affordability (i.e., whether the farmer is able to pay for the vaccine). The variables contributing to “knowledge of vaccines and animal health” included the extent of knowledge of animal health and access to information on PPR vaccination.

**Figure 5:**
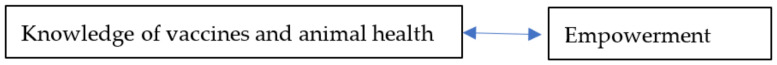
Illustration of the PLS-SEM model results on the relationship between knowledge (of vaccines and animal health) and empowerment.

The results indicate that the links between knowledge (of PPR vaccines and animal health) and empowerment are highly significant (*p* < 0.05) for both women (*β* = 0.11, *p* = 0.045) and men (*β* = 0.32, *p* = 0.000). This implies that, for both women and men, knowledge of PPR vaccines and animal health is strongly correlated with empowerment (Figure 5). Consequently, even though no causality can be established by this model’s results, chances are significantly high that more knowledgeable farmers, in terms of animal health and PPR vaccine, are also empowered.

**Figure 6:**
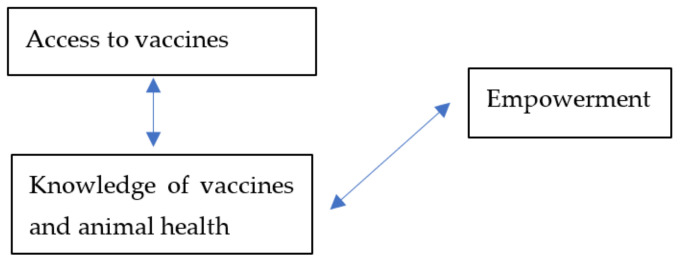
Illustration of the PLS-SEM model results on the relationship between access to vaccines and empowerment.

Further, the results (Table 4 and Table 5) show that participation in vaccination and access to vaccines are not significantly directly linked with empowerment. In other words, the results reveal that one cannot predict empowerment from the access and participation variables or the other way round. However, the results reveal that being able to access vaccines or accessing vaccines has an indirect association with empowerment since access is positively correlated with knowledge of vaccines and animal health, which, in turn, is positively correlated with empowerment (Figure 6). These results were similar for both men and women goat farmers. For both men and women, a significant mediation effect of “knowledge of vaccines and animal health” on the relationship between access to vaccines and empowerment was evident. This implies that, for men and women, access to vaccines exhibits an important relationship with empowerment because of its indirect significant relationship, i.e., knowledge of vaccines and animal health is an intervening facet in the relationship between access to vaccines and empowerment.

**Figure 7:**
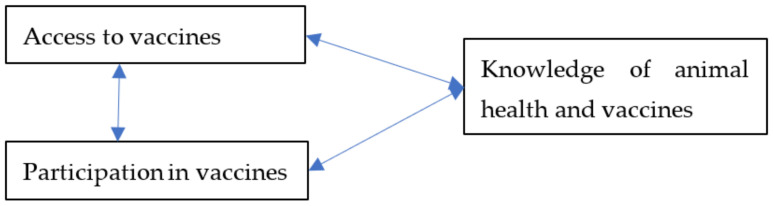
Illustration of the PLS-SEM model results on the relationship between vaccination facets for women.

Moreover, while participation in vaccination is not significantly linked to empowerment, it is strongly linked to access to vaccines and knowledge of vaccines and animal health for women (Figure 7).

**Figure 8:**
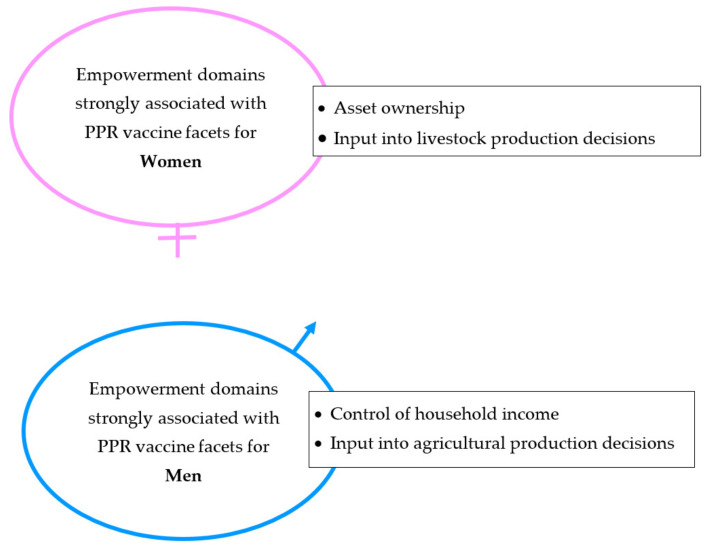
Illustration of the PLS-SEM model results on the empowerment dimensions that were strongly associated with the PPR vaccine facets.

Exploring the linkage between vaccine facets and empowerment, the model results (Table 4 and Table 5, also illustrated in Figure 8) revealed that there is a difference between men and women respondents in terms of the latent construct, i.e., the empowerment measure constructed from the domains of empowerment (WELI). For women, “asset ownership” and “input into decisions concerning livestock production” (two WELI indicators) are the empowerment domains that are strongly associated with the facets of PPR vaccine. For men, empowerment values are strongly and significantly represented by “control of income” and “input into decisions concerning agricultural production” indicators.

## 4. Discussion

We analyzed the linkage between WE and vaccination facets in the context of PPR vaccine interventions in rural Ghana. Empowerment was measured within the context of livestock production—a more general measure of empowerment, such as using the WEAI instead of WELI, might have had a weaker association in such a study. Our study findings reveal the specific WELI dimensions that significantly correlate with vaccination facets.

Our results demonstrate a strong positive association between “knowledge about vaccines and animal health” and empowerment for both women and men. It is worth noting here that though ‘“knowledge’” seems to go beyond knowledge of only the vaccine since it also included knowledge of animal health, this knowledge of animal health was mapped in the context of PPR. The association between knowledge and empowerment is widely explored in the literature on agricultural development. A significant relationship between the empowerment status of women and farming experience (a proxy of knowledge) [84] and a connection between women’s access to knowledge and empowerment in seed systems [85] has been observed. Two dimensions of empowerment were found to be relevant in East Africa (Kenya, Uganda and Tanzania), one is associated with knowledge [86]. A number of studies further connect knowledge and the related increase in empowerment to other development outcomes, such as animal productivity [7], household food security [87,88,89,90], and the health status of children [91].

Our study found that “participation in vaccination” had no direct link to empowerment for both men and women, though it was significantly linked to “knowledge of vaccines and animal health”. In the study area, men rather than women take the responsibility of procuring veterinary services [92]. Consequently, the variables that significantly define ‘“participation in vaccination’” (Table 4 and Table 5) encompass actions normatively within the men’s domain. One would assume “participation” increases “knowledge” and therefore empowerment. However, this was not evident in our results. One possible explanation is that providing labor and other resources necessary to get one’s animals vaccinated (for instance, taking the animals to be vaccinated or going to source the vaccines/vaccinators and/or paying for costs that may be incurred in the vaccination process) may result in an increased labor and financial burden in ways that do not improve empowerment. From a WE perspective, work overload has been found to be a constraining factor on WE and agricultural productivity [93,94]. Consequently, interventions that aim to increase the access of women to animal vaccines by enhancing women’s participation in the vaccination process itself may increase women’s work burden with negative consequences on their empowerment.

Our results also found that “access to vaccines” exhibited an indirect relationship with empowerment, mediated by “knowledge about vaccines and animal health”. This observation implies that one cannot predict changes in empowerment by observing access to vaccines alone. The model results indicate that having access to vaccine suppliers and being financially able to buy the products has no direct links with empowerment, but a link can be observed indirectly through “knowledge about vaccines and animal health”. Policies aimed at making the animal vaccination system more gender-responsive should therefore consider women’s access to vaccines and knowledge about them as strongly connected with each other in their relation to empowerment. This argument is supported by [95,96], who argue that livestock keepers’ access to education and advice from trained professionals (proxies of “knowledge”) and access to legitimate veterinary medicinal products (a proxy of “access to vaccines”) are both essential components of effective animal health services and are of fundamental importance to their enlightenment and decision-making.

Our findings show a significant difference in the domains of empowerment that were significantly associated with the facets of PPR vaccines between men and women in goat-keeping households. For men, the empowerment domains significantly associated with the vaccine facets were “control of income” and “input into decisions concerning general agricultural production” indicators. For women, “asset ownership” and “input into decisions concerning livestock production” are the domains of empowerment that were found to be significantly associated with PPR vaccine facets. These results corroborate, along with evidence obtained worldwide, the importance of ownership over productive resources and decision-making in agricultural productivity for WE. In Pakistan, lack of control over income and lack of control over resources were found as the domains that contribute most to women’s disempowerment [97]. For Ghanaian women in crop-oriented agriculture, [98] found that control over use of income, asset ownership, and access to and decisions concerning credit were the main sources of women’s disempowerment.

Specifically for livestock, it is argued that it is important that women own productive assets and are able to make important decisions in livestock value chains, since participation alone may not benefit women [99]. This is because women in sub-Saharan Africa contribute substantially to livestock production (with variations depending on the species), while their ability and power to take decisions over the livestock enterprise is often limited even for species they own [16]. Gender norms affecting livestock ownership were observed in northern Ghana, where it is largely seen as disrespectful for a married woman to say that she owns livestock [92]. In Ethiopia women can own some livestock but need their husband’s approval and support to take them to the market [100]. It is therefore not surprising that the connection between WE and goat vaccination is evident through women’s asset ownership and decision making over livestock production. Reducing female–male differences in resource access and leveraging female–male differences to increase women’s relative decision-making authority related to livestock management have been found necessary for vaccine uptake [8].

The fact that the domains of men’s empowerment mostly associated with goat vaccination were “control of income” and “input into decisions about general agricultural production” is somehow less straightforward. Possibly, because men in Ghana are the ones generally considered responsible for earning and controlling household income and for getting veterinary services [92], they would be constrained if they lacked control of household income and decisions concerning agricultural production (a source of income). Alternatively, it is possible that the findings refer to men’s engagement in agriculture vis-à-vis men who engage in non-agricultural activities. In other words, in cases where men control income and make decisions about agricultural production, i.e., where they are involved in agricultural activities, they are more likely to have knowledge about vaccines and animal health, access to vaccines, and/or to participate in getting their household’s livestock vaccinated, given that men have much easier access to vaccines in Ghana than women.

Qualitative fieldwork needs to be conducted to better explore the complexities of these associations and answer questions such as “Do men lack control over household income?”, “Is it the women who have control over household income?”, “Do men have less participation in decision-making on agricultural production vis-à-vis women while women lack decision-making with regard to livestock?”. Since gender is not the only factor (but an intersection of several factors that illuminate different barriers to vaccine uptake [101]), further investigations into the status of the livestock production system and household dynamics in the study area would be necessary to provide this important additional information to enhance understanding of existing practices and norms. This would, in addition to our findings, contribute to informing a better formulation of a more equitable PPR vaccine system not only in Ghana but also in many low- and middle-income countries with similar production systems and challenges. Overall, these findings indicate that livestock interventions that aim to support empowerment within the framework of global eradication of PPR (i.e., establishing more effective and equitable PPR vaccine systems) would benefit from some strategic focus on enhancing “asset ownership” and “input into decisions concerning livestock production” for women and “control of income” and “input into decisions concerning agricultural production” for men.

Since structural equation models (SEM) do not aim to establish causal relations from associations alone (i.e., SEM is an inference engine that takes in two inputs, qualitative causal assumptions and empirical data, and derives two logical consequences of these inputs: quantitative causal conclusions and statistical measures of fit for the testable implications of the assumptions), fitting the data does not “prove” the causal assumptions but it makes them somewhat more plausible [102]. Consequently, the direction of causality cannot be determined in this cross-sectional study. We therefore recommend that the causal pathway between WE and animal vaccination be established. This will be possible in the longitudinal study for which the current data set provides the baseline. That notwithstanding, the results show that taking advantage of the synergies offered by empowering women and supporting their access to and knowledge of vaccinations can greatly enhance vaccination rates, supporting improvements in livestock productivity and household livelihoods. To this aim, our results imply the need for livestock interventions to strategically support the empowerment of women by enhancing asset ownership, input into decisions regarding livestock production, knowledge of vaccines and animal health, and access to vaccines.

## 5. Conclusions

The majority of households in northern Ghana keep small ruminants, mainly goats [45]. Although women have greater control over goats [44], vaccine delivery systems favor men. Therefore, the devastating effects of PPR or “goat plague” could have comparatively larger effects on women. Gender-blind animal health interventions are incapable of addressing the gendered constraints embedded in livestock systems and will continue to fail to eradicate deadly diseases, including PPR, in livestock kept by women in the affected countries [101]. It is therefore important that the PPR eradication efforts, particularly strategies for strengthening veterinary services, better respond to gender-based constraints and opportunities and intentionally support WE in a bid to find the most effective approach for the disease’s global eradication.

Small ruminants are among the species that are considered key to support the empowerment of rural women because they can be purchased and controlled by women, can be used by women to accumulate wealth—in the absence of other financial institutions—and can be easily liquified if cash is needed to deal with an emergency. Yet women in Ghana (as in other countries in Africa [103]) have limited access to vaccines, including for PPR, that are essential for the health and productivity of their livestock. Understanding the social constraints that limit women’s access to and participation in PPR vaccinations is critical for informing the global strategy for the eradication of PPR [32]. This study analyzed the correlation between WE and PPR vaccination facets using data from goat keeping households in northern Ghana. From the results, we established that “access to vaccines” is strongly associated with “knowledge of animal health and vaccines”, and the latter is strongly associated with WE. We also established that “asset ownership” and “input into decisions concerning livestock production” are two key domains in the association between the PPR vaccines facets and WE among women goat keepers in the study area.

Since the direction of causality cannot be determined in this cross-sectional study, the study therefore recommends that the causal pathways (beyond correlation) between WE and PPR vaccination are investigated in future studies. Even so, the observed correlation between WE and vaccination facets in this study, based on other empirical evidence from the literature on the ways in which animal vaccine facets may affect WE and vice versa, the results of the study yield useful implications for the design of gender-responsive animal health delivery systems. The study’s striking result is that the empowerment dimensions that are strongly associated with PPR vaccine facets differ for men and women. Consequently, the design of gender-responsive animal health delivery systems needs to take such differences into account. In particular, such animal health delivery interventions would need to consider the effects, ways, and means of supporting women’s access to household assets (including livestock) and women’s involvement in household decisions concerning livestock production while considering men’s control of household income and input into agricultural activities. This result could be useful in informing gender transformative and gender accommodative approaches (GTAs and GAAs). Our research results also reinforce, empirically, the intuitive perception of the relevance of “knowledge of animal health and vaccines” and “access to vaccines”, two factors that would be useful in informing both GTAs and GAAs in designing gender-responsive animal health delivery systems.

This research on the relationship between WE in livestock and vaccination facets focuses on a single developing country. Future research could extend the work in different ways, such as by examining whether similar findings are to be observed in different developing countries with livestock species where there are differences in gender norms, policies, and animal vaccination systems that affect the involvement of women in livestock production decision making.

## Figures and Tables

**Table 1:** Vaccine interventions variables from WELI Ghana data.

The Vaccine Module Questions Were Classified into Questions That Observe		Variable Description	Variable Name (Used in the PLS-SEM Model)
Knowledge and attitude about vaccines and animal health	Where to buy	Knowing where to get PPR vaccines	Know_where_get
	Level of knowledge (low, medium, high)	Know government’s role in vaccinations	Know_regulation_govt_role
		Knowing vaccine regulations	Know_regulation
		Knowing who can vaccinate	Know_regulation_vaccinate
		Knowing about animal health	Know_vaccine_health
	Vaccines use	Knows the best time to administer PPR vaccines	Know_vaccine_administer
	Attitude	Would like to access suppliers;	Aspire_access_suppliers
		Think vaccines can prevent PPR	Attitude_vaccines_can
	Impact of vaccinations	Level of severity of past infection in the herd, by extent of loss of animals	Impact_loss_animals
	Access to information about vaccines and animal health	Attending training and seminars on animal health	Attend_training
		Access to training/seminars on small ruminants’ health	Access_info_training
		Access to information on PPR vaccination	Access_info_vaccine
Participation in vaccinations	Purchasing	Who participated in purchasing PPR vaccines	Who_purchase_vaccine
		Woman participated in purchasing PPR vaccines, either alone or with others	Woman_purchase_vaccine
		Man participated in purchasing PPR vaccines, either alone or with others	Man_purchase_vaccine
	Paying for vaccination	Man paid for vaccine	Man_pays_vaccine
		Woman paid for vaccine	Woman_pays_vaccine
		Both man and woman paid for PPR vaccine	Man_woman_pays_vaccine
		Who pays for PPR vaccines	Who_pays_vaccine
		Woman pays for PPR vaccines either singly or with men	Woman_s_j_pays_vaccine
		Man pays for PPR vaccines, either singly or with woman	Man_s_j_pays_vaccine
	Vaccinating	One’s goats have been vaccinated against PPR	Vaccinated_PPR
		Woman actively participates in the process of vaccinating animals against PPR	Woman_vaccinate_PPR
		Man actively participates in the process of vaccinating animals against PPR	Man_vaccinate_PPR
Access to vaccine	Physical access	Access to PPR vaccine suppliers/vaccinators	Access_suppliers
		Access to cold chain for PPR vaccine supplies	Access_cold_chain
	Affordability	Ability to pay for PPR vaccine/vaccination	Afford_vaccine

**Table 2:** Sociodemographic characteristics of respondents and their households.

Respondent and Household Characteristics		Women Value (*n* = 465)	Men Value (*n* = 92)	*t*-Test
Household structure	Age of respondent age (mean age)	44.14	46.34	1.47, df = 555
	Household size (mean number or persons)	6.44	7.65	4.48, df = 555 ***
Percentage of respondents who felt the livestock species (out of the species kept in the household) to be most important for their household’s livelihood	Small ruminants (sheep and/or goats—local or improved breeds)	59.35	30.43	-
	Chickens (local or improved breeds)	10.54	17.39	-
	Large ruminants e.g., cattle (beef or dual-purpose—local or improved breeds)	18.71	30.43	-
	Large ruminants e.g., cattle (dairy—local or improved breeds)	10.75	19.57	-
	Pigs (and/or others—local or improved)	0.65	2.17	-
Percentage of respondents who felt the livestock species (out of the species kept in the household) to be most important for their own livelihood	Small ruminants (sheep and/or goats—local or improved breeds)	46.45	53.26	-
	Chickens (local or improved breeds)	42.58	32.61	-
	Large ruminants e.g., cattle (beef or dual-purpose—local or improved breeds)	0.86	8.70	-
	Large ruminants e.g., cattle (dairy—local or improved breeds)	1.51	5.43	-
	Pigs (and/or others—local or improved)	8.60	0.00	-

Note: *** denotes significant difference at 1% confidence level; df denotes degrees of freedom.

**Table 3:** Reliability and validity tests.

Constructs	Variable Name in the Data	Women					Men				
		λ	CA	rho_A	CR	AVE	Λ	CA	rho_A	CR	AVE
Access to vaccines	Access_suppliers	0.82	0.66	0.70	0.85	0.74	0.81	0.69	0.76	0.86	0.76
	Afford_vaccine	0.90					0.92				
Knowledge of vaccines and animal health	Access_info_vaccine	0.93	0.74	0.80	0.88	0.79	0.91	0.79	0.79	0.91	0.83
	Know_vaccine_health	0.85					0.91				
Participation in vaccination	Vaccinated_PPR	0.94	0.81	0.89	0.88	0.73	0.92	0.72	0.84	0.84	0.65
	Who_pays_vaccine	0.95					0.92				
	Who_purchase_vaccine	0.64					0.53				
WELI	Asset_ownership	0.73	0.26	0.26	0.73	0.57	-	-	-	-	-
	Input_decisions_livestock	0.78					-				
	Input_decisions_agric	-	-	-	-	-	0.84	0.45	0.46	0.78	0.64
	Control_income	-					0.77				

Note: λ denotes factor loadings; CA denotes cronbach’s alpha; CR denotes composite reliability; AVE denotes average variance extracted.

**Table 4:** Direct relationships (hypothesis) and mediation analysis (standardized path coefficients of latent variables for overall sample) for women.

Hypotheses	Original Sample (O) (*n* = 465)	Sample Mean (M) (*n* = 5000)	Standard Deviation (STDEV)	*p*-Values
Access to vaccines <> knowledge of vaccines and animal health	0.22	0.22	0.05	0.000
Access to vaccines <> participation in vaccination	0.62	0.62	0.03	0.000
Access to vaccines <> empowerment	0.09	0.09	0.06	0.126
Knowledge of vaccines and animal health <> participation in vaccination	0.29	0.29	0.05	0.000
Knowledge of vaccines and animal health <> empowerment	0.11	0.12	0.06	0.045
Participation in vaccination <> empowerment	0.06	0.06	0.07	0.378

Note: <> denotes the relationship between the variables, implying correlation rather than causation.

**Table 5:** Direct relationships (hypothesis) and mediation analysis (standardized path coefficients of latent variables for overall sample) for men.

Hypotheses	Original Sample (O) (*n* = 97)	Sample Mean (M) (*n* = 1000)	Standard Deviation (STDEV)	*p*-Values
Access to vaccines <> knowledge of vaccines and animal health	0.48	0.48	0.07	0.000
Access to vaccines <> participation in vaccination	0.55	0.56	0.08	0.000
Access to vaccines <> empowerment	0.09	0.10	0.13	0.483
Knowledge of vaccines and animal health <> participation in vaccination	0.14	0.14	0.09	0.141
Knowledge of vaccines and animal health <> empowerment	0.32	0.33	0.09	0.00
Participation in vaccination <> empowerment	0.06	0.05	0.10	0.516

Note: <> denotes the relationship between the variables, implying correlation rather than causation.

## Data Availability

The data presented in this study are available on request from the corresponding author. The data are not publicly available since the project under which the data was collected and various related data collection activities are still on-going.

## Appendix A

**Table A1:** WELI indicators.

Agency Classification	Indicators (WELI Subdimension)	Definition
Intrinsic Agency (Power within)	Autonomy in income	More motivated by own values than by coercion or fear of others’ disapproval: Relative Autonomy Index score >= 1 RAI score is calculated by summing responses to the three vignettes about a person’s motivation for how they use income generated from agricultural and non-agricultural activities (yes = 1; no = 0), using the following weighting scheme: 0 for vignette 1 (no alternative), 2 for vignette 2 (external motivation), 1 for vignette 3 (introjected motivation), and +3 for vignette 4 (autonomous motivation)
	Self-efficacy	New General Self-Efficacy Scale: ‘‘Agree” or greater on average with eight self-efficacy questions
	Attitudes about intimate partner violence against women	Believes husband is NOT justified in hitting or beating his wife in all 5 scenarios: If: (1) She goes out without telling him (2) She neglects the children (3) She argues with him (4) She refuses to have sex with him (5) She burns the food
	Respect among household members	Meets ALL of the following conditions related to their spouse, the other respondent, or another household member: (1) Respondent respects relation (most of the time) (2) Relation respects respondent (most of the time) (3) Respondent trusts relation (most of the time) (4) Respondent is comfortable disagreeing with relation (most of the time)
Instrumental agency (Power to)	Input in productive decisions—general:	Meets at least ONE of the following conditions for ALL of the agricultural activities they participate in: (1) Makes related decision solely (2) Makes the decision jointly and has at least some input into the decisions (3) Feels could make decision if wanted to (to at least a MEDIUM extent)
	Input in productive decisions–livestock:	Meets at least ONE of the following conditions for ALL of the livestock activities they participate in: (1) Makes related decision solely (2) Makes the decision jointly and has at least some input into the decisions (3) Feels could make decision if wanted to (to at least a MEDIUM extent)
	Ownership of land and other assets	Owns, either solely or jointly, at least ONE of the following: (1) At least THREE moveable assets (equipment or consumer durables) (2) Land
	Access to and decisions regarding financial services	Meets at least ONE of the following conditions: (1) Belongs to a household that used a source of credit in the past year AND participated in at least ONE sole or joint decision about it (2) Belongs to a household that did not use credit in the past year but could have if wanted to from at least ONE source (3) Has access, solely or jointly, to a financial account
	Control over use of income	Has input in decisions related to how to use BOTH income and output from ALL of the agricultural activities they participate in AND has input in decisions related to income from ALL non-agricultural activities they participate in, unless no decision was made
	Work balance	Works less than 10.5 h per day: Workload = time spent in primary activity + (1/2) time spent in childcare as a secondary activity
	Ability to visit important locations	Meets at least ONE of the following conditions: (1) Visits at least TWO locations at least ONCE PER WEEK of (city, market, family/relative); or (2) Visits at least ONE location at least ONCE PER MONTH of (health facility, public meeting)
Collective agency (Power with)	Group membership	Active member of at least ONE group
	Membership in influential groups	Active member of at least ONE group that can influence the community to at least a MEDIUM extent
